# Effects of Long-Term Administration of Bovine Bone Gelatin Peptides on Myocardial Hypertrophy in Spontaneously Hypertensive Rats

**DOI:** 10.3390/nu15245021

**Published:** 2023-12-06

**Authors:** Songmin Cao, Xinyu Wang, Lujuan Xing, Wangang Zhang

**Affiliations:** 1 School of Food Science and Engineering, Ningxia University, Yinchuan 750021, China; songmin_cao@nxu.edu.cn (S.C.); song90000@126.com (X.W.); 2 Key Lab of Meat Processing and Quality Control, MOE, College of Food Science and Technology, Nanjing Agricultural University, Nanjing 210095, China; xinglujuan@163.com

**Keywords:** bovine bone gelatin peptides, myocardial hypertrophy, myocardial fibrosis, proteomics, Pi3k/Akt

## Abstract

The research purpose was to investigate the effects and the underlying molecular mechanisms of bovine bone gelatin peptides (BGP) on myocardial hypertrophy in spontaneously hypertensive rats (SHR). BGP relieved myocardial hypertrophy and fibrosis in SHR rats in a dose-dependent manner by reducing the left ventricular mass index, myocardial cell diameter, myocardial fibrosis area, and levels of myocardial hypertrophy markers (atrial natriuretic and brain natriuretic peptide). Label-free quantitative proteomics analysis showed that long-term administration of BGP changed the left ventricle proteomes of SHR. The 37 differentially expressed proteins in the high-dose BGP group participated in multiple signaling pathways associated with cardiac hypertrophy and fibrosis indicating that BGP could play a cardioprotective effect on SHR rats by targeting multiple signaling pathways. Further validation experiments showed that a high dose of BGP inhibited the expression of phosphoinositide 3-kinase (Pi3k), phosphorylated protein kinase B (p-Akt), and transforming growth factor-beta 1 (TGF-β1) in the myocardial tissue of SHR rats. Together, BGP could be an effective candidate for functional nutritional supplements to inhibit myocardial hypertrophy and fibrosis by negatively regulating the TGF-β1 and Pi3k/Akt signaling pathways.

## 1. Introduction

Pathological myocardial hypertrophy is a common pathological process of valvular heart disease, cardiomyopathy hypertension, and other cardiovascular diseases [1]. Myocardial hypertrophy, as a compensatory response of myocardial tissue to increased cardiac load, is an independent risk factor for cardiovascular diseases [2]. Pathological myocardial hypertrophy is mainly manifested as cardiomyocyte hypertrophy, left ventricular wall thickening, increased cardiac mass, and cardiac systolic and diastolic dysfunction of the heart [3]. Normally, pathological myocardial hypertrophy could induce excessive accumulation of extracellular matrix proteins (e.g. type I and type III collagen) in the myocardium, and ultimately lead to the occurrence of myocardial fibrosis [4]. As one of the complicated health problems with a high rate of morbidity and mortality, pathological myocardial hypertrophy could result in heart failure and sudden death [5]. Therefore, preventing and delaying the progression of myocardial hypertrophy and myocardial fibrosis may be one of the effective treatment strategies to prevent and treat heart failure. The pathogenesis of pathological myocardial hypertrophy and fibrosis mainly involves two factors: biomechanical stress and neurohumoral factors. Among them, biomechanical stress mainly refers to mechanical overload [6]. Neurohumoral factors include sympathetic nerve excitation, renin-angiotensin-aldosterone system (RAS), insulin/insulin receptor/Akt signaling pathway, β-adrenergic receptor signaling pathway, Ca^2+^/calmodulin-dependent kinase II signaling pathway, phosphoinositide 3-kinase/protein kinase B (Pi3k/Akt) signaling and various cytokines (such as transforming growth factor-beta 1 (TGF-β1), endothelin-1, tumor necrosis factor) [7,8,9]. According to different targets and pathways, commonly used drugs for curing myocardial hypertrophy include angiotensin-converting enzyme inhibitors, diuretics, beta-receptor blockers, angiotensin receptor inhibitors, and calcium channel blockers [10]. Although these drugs can reduce or reverse the development of left ventricular hypertrophy to a certain extent, long-term administration could cause some side effects [11]. Thus, researchers are increasingly committed to developing safer, economical, and effective natural materials for cardiac protection.

Bioactive peptides usually contain 2–20 amino acid residues and some of them have preventive and therapeutic effects on many chronic diseases [12]. Studies have shown that many proteins can release biologically active peptides that are beneficial for cardiovascular therapy [13]. Among them, collagen has received widespread attention because of its sustainable source, excellent bioavailability, and neutral taste [14,15]. Over the past decade, the study of collagen peptide has focused on its anti-hypertensive, anti-oxidant, and anti-diabetic effects [14]. However, there are few studies investigating the role and mechanism of collagen peptides against myocardial hypertrophy and myocardial fibrosis. In our previous studies, we have found that bovine bone gelatin peptide (BGP) had significant effects on reducing blood pressure in spontaneous hypertension (SHR) rats [13]. Long-term elevated blood pressure can induce cardiac hypertrophy in patients, and it has been proven that controlling blood pressure can prevent and alleviate the occurrence and development of cardiac hypertrophy [16]. Thus, this study was conducted to investigate the reversal and therapeutic effects of BGP on hypertension-induced cardiac hypertrophy/fibrosis and its underlying molecular mechanisms.

## 2. Materials and Methods

### 2.1. Materials

Bovine bone gelatin extracted by our laboratory; Trypsin (Promega Biotech Co., Ltd., Madison, WI, USA); Alcalase 2.4 L (Novozymes Biotechnology Co., Ltd., Tianjin, China); DTT (Absin Bioscience Inc., Shanghai, China); Rabbit polyclonal to PP1C gamma (anti-Ppp1cc) (Abcam, Cambridgeshire, UK); Rabbit monoclonal [EP817Y] to Glycogen synthase 1 (anti-Gys1) (Abcam, Cambridgeshire, UK); Rabbit polyclonal to EEF2 (anti-Eef2) (Abcam, Cambridgeshire, UK); PI3 Kinase p85 (19H8) rabbit mAb (anti-Pi3k) (Cell signaling technology (CST), Boston, MA, USA); Akt (pan) (C67E7) rabbit mAb (anti-Akt) (CST, Boston, MA, USA); Phospho-Akt (Ser473) (D9E) XP^®^ Rabbit mAb (p-Akt) (CST, Boston, MA, USA); anti-GADPH (Thermo, Waltham, MA, USA); Goat anti-rabbit IgG (Biogot technology, Co., Ltd., Nanjing, China); Goat anti-mouse IgG (Biogot technology, Co., Ltd., Nanjing, China).

### 2.2. Preparation of Bovine Bone Gelatin Peptides

Bovine bone gelatin solution was prepared with ultrapure water (8:100, *w/v*) at 55 °C. Then, the pH value of the bovine bone gelatin solution was adjusted to 9.0 by adding 2 M NaOH. Subsequently, Alcalase 2.4 L (7200 U/g) was used to hydrolyze the bovine bone gelatin solution at 55 °C for 6.5 h. Finally, the hydrolysate was boiled for 20 min to inactivate Alcalase 2.4 L. After the hydrolyzate was cooled to room temperature, its pH value was adjusted to 7.0 with 1 M HCl. The large molecules in the gelatin hydrolysate were removed with a 3 kDa molecular weight (MW) cut-off membrane. Freeze-dried bovine bone gelatin peptides (MW ≤ 3 kDa, BGP) were collected and stored at −80 °C for subsequent experiments. The specific mixed peptide sequences in BGP are shown in Supplementary Table S1 [17].

### 2.3. Animal Experiment and Sample Collection

All experimental animals (spontaneously hypertensive rats: SHR, *n* = 48, male, 9 weeks old, 225 ± 20 g, SBP > 180 mmHg; Wistar-Kyoto rats: WKY, *n* = 12, male, 9 weeks old, 225 ± 20 g, normotensive) were purchased from Beijing Weitong Lihua Experimental Animal Technology Co., Ltd. Sixty experimental rats were housed in separate cages and placed in a room with an ambient humidity of 35% (22 ± 2 °C, 12 h light/dark cycle) for 1 week. One week later, those rats were randomly divided into five experimental groups with each group containing 12 rats. WKY (*n* = 12) and SHR rats (*n* = 12) treated with 0.9% saline per day were set as negative control groups for normal blood pressure (WKY-NCG) and hypertension (SHR-NCG), respectively. SHR rats (*n* = 12) treated with captopril (10 mg/kg BW) per day were set as positive control group (SHR-PCG). SHR rats (*n* = 12) treated with low-dose BGP (SHR-LPG, 100 mg/kg BW) and high-dose BGP (SHR-HPG, 200 mg/kg BW) were set as experimental groups. The BGP and captopril used for the treatment of rats were dissolved in a 0.9% NaCl solution and then orally administered to the rats through a gastric cannula for 10 weeks.

After 10 weeks, the body weight of each rat (BW) was recorded. Then, the experimental animals anesthetized by isoflurane were sacrificed through abdominal aortic bleeding. Pre-cooled phosphate buffer solution was used to wash the excess blood on the extracted heart, and then the whole heart wet weight (HW) and left ventricular wet weight (LVW) were measured with an analytical balance. The rat’s HW and LVW were used to calculate the rat’s heart mass index (HMI, HMI = HW/BW) and left ventricular mass index (LVWI, LVWI = LVW/BW). Finally, the left ventricle tissue of rats and serum were minced and stored at −80 °C for further molecular biology analysis. The whole heart washed with pre-cooled phosphate buffer solution was fixed with a 4% neutral formalin solution for further histological analysis.

### 2.4. Histological Measurement

The whole hearts of 6 rats in each treatment group were randomly selected for histological measurement. In order to assess the degree of cardiomyocyte hypertrophy and cardiac fibrosis in rats, the hearts soaked in 4% formalin solution were taken out and embedded in paraffin according to standard histological methods [5]. Subsequently, these hearts were sliced into 6 μm thick sections with a microtome (Leica Microsystems, Nussloch, Germany), and then HE or Masson staining was performed on these sections. A digital camera system (CX41, Olympus, Singapore) was used to obtain micrographs. A digital image analysis system (Image-Pro Plus 6.0) was used to measure the diameter and fibrosis area of cardiomyocytes.

### 2.5. Label-Free Proteomics Analysis

The left ventricular tissues of 6 rats in each treatment group were randomly selected for proteomics analysis. The pre-cooled RIPA lysis buffer (with protease inhibitor and phosphatase inhibitor) was used to extract the whole protein in the left ventricular myocardial tissue of rats for subsequent digestion operations [18]. The concentration of extracted protein was determined using a BCA protein detection kit (Thermo, Shanghai, China). First, the protein sample (300 μg) was reduced with DTT at 60 °C for 1 h. Then, 10 mM iodoacetamides were used to alkylate the reduced protein at 23 °C in the dark for 45 min. Next, the protein sample was diluted with NH_4_HCO_3_ (50 mM) and incubated with trypsin (1:50 *w/w*) at 37 °C for 16 h. Subsequently, the polypeptide solution digested with trypsin was collected and acidified with 10% formic acid. A C18 ZipTip pipette tip was used for desalting the peptide solution. After vacuum freeze-drying, the digested solution was stored for further analysis by high-performance liquid chromatography-tandem mass spectrometry (HPLC-MS/MS, ThermoFisher Scientific C Ltd., Waltham, MA, USA).

HPLC-MS/MS was used to analyze the lyophilized digestion solution resuspended in 0.2% formic acid buffer. Briefly, the sample (loading volume: 5 μL, flow rate: 8 μL/min) was loaded onto a C18 column (3 μm, 100 μm × 20 mm) using the easy nano-LC sampling system. After that, the peptides attached to the chromatographic column were eluted to the C18 analytical chromatographic column (1.9 μm, 120 mm × 150 μm). Then, the separated peptides were identified and analyzed by the LTQ-Orbitrap XL mass spectrometer. The data file detected by the orbital ion trap was converted into a graphic file (MGF) by Proteome Discoverer 1.2 (5600 msconverter) for the collection and identification of protein information.

The mass spectrum information obtained by HPLC-MS/MS analysis was analyzed by Proteome Discoverer software 2.0 (Thermo Fisher Scientific, Waltham, MA, USA). The Sequest HT engine was used to identify the protein based on the Uniprot RAT database. Peptide false discovery rate (FDR) ≤ 0.01, fold change (FC) <0.67 or >1.5, and *p*-value < 0.05 were set as the screening criteria for differential proteins. Differential protein function and metabolic pathway analysis were explored using gene ontology (GO) enrichment and Kyoto Encyclopedia of Genes and Genomes (KEGG) pathway enrichment. The protein-protein interaction (PPI) analysis was carried out through STRING (http://string-db.org/, accessed on 1 November 2020).

### 2.6. Western Blotting

The expression levels of Ppp1cc, Gys1, Eef2, Pi3k, Akt, and phospho-Akt (p-Akt) in rat myocardium were determined by Western blotting analysis. The RIPA lysate buffer including protease inhibitor and phosphatase inhibitor was used to extract the total protein in the left atrium myocardium of rats. A 10% SDS-polyacrylamide gel was used to separate 40 μg total protein samples at 100 V. The separated target protein bands were transferred to the polyvinylidene fluoride (PVDF) membrane (0.22 μm, Millipore, Billerica, MA, USA) under a constant voltage of 90 V. After the transfer, the PVDF membrane was sealed with 5% skimmed milk buffer (dissolved in TBST containing 0.1% Tween 20). Then, the corresponding primary antibody was incubated with the blocked PVDF membrane at 4 °C for 14 h. After the primary antibody incubation, the washed PVDF membrane was incubated with the corresponding secondary antibody at a dilution factor of 1:5000 for 2 h. After incubation, the Image Quant LAS4000 (GE, Fairfield, CT, USA) was used to visualize the protein bands on the PVDF membrane. The Quantity-One 4.6.2 software was used to quantify the band signal intensity, and the protein expression level was standardized to the corresponding GAPDH level. The dilution ratios of anti-GADPH, anti-Ppp1cc, anti-Eef2, anti-Gys1, anti-Pi3k, anti-Akt, and anti-p-Akt were 1:5000, 1:1000, 1:2000, 1:5000, 1:1000, 1:2000, and 1:1000, respectively. 

### 2.7. Enzyme-Linked Immuno Sorbent Assay (ELISA)

The expression levels of myocardial hypertrophy markers (brain natriuretic peptide (BNP), atrial natriuretic peptide (ANP)) and TGF-β1 in rat serum and myocardial tissue were measured with Elisa kit purchased from Jiangsu Meimian Industrial Co., Ltd (Yancheng, Jiangsu, China).

### 2.8. Statistical Analysis

All experiments were repeated 6 times separately, and all the experimental results for presented by mean ± standard error (SE). SPSS 25.0 software (IBM SPSS Inc., Chicago, IL, USA) was used for a one-way ANOVA statistical analysis of experimental results. Duncan’s multi-range test was used for multiple comparisons (*p* < 0.05 indicating significant difference and *p* < 0.01 indicating extremely significant difference).

## 3. Results

### 3.1. Effects of BGP on Myocardial Hypertrophy and Myocardial Fibrosis in SHR

**Figure 1:**
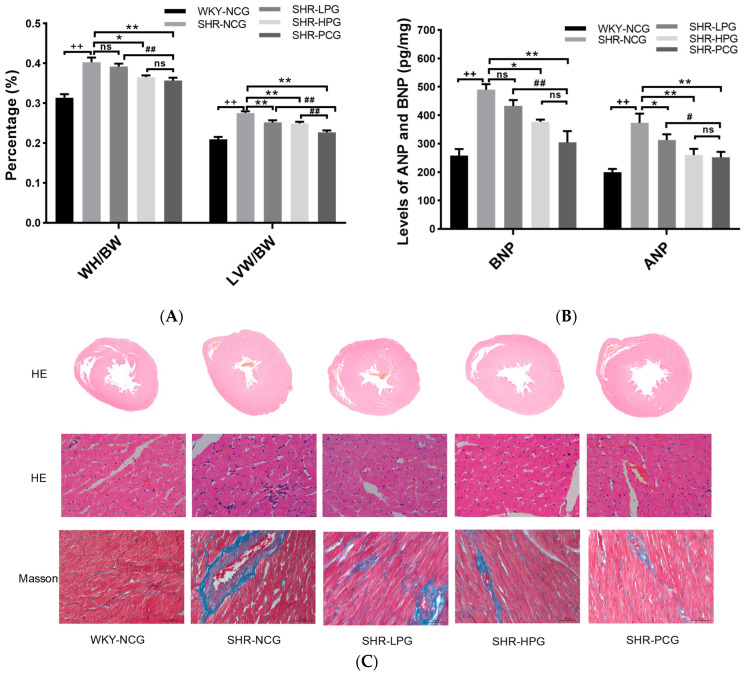

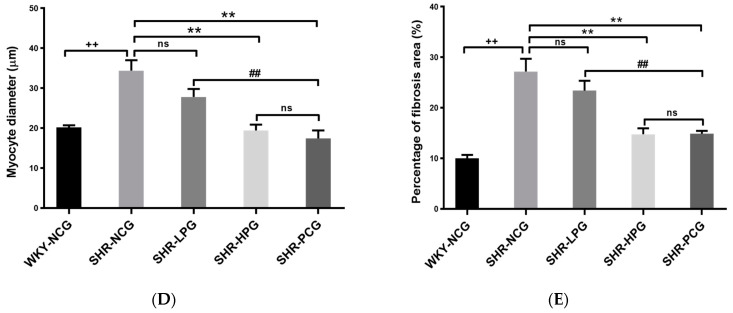
Effects of long-term oral BGP on myocardial hypertrophy and fibrosis in SHR rats. Note: (**A**) Heart weight index (HMI) and left ventricular mass index (LVWI) of rats; (**B**) The levels of ANP and BNP of rats; (**C**) HE and Masson staining of the left ventricle of rats with the magnification of 1×, 400×, 400× respectively; (**D**) Cardiomyocyte diameter of rats; (**E**) Percentage of myocardial fibrosis area of rats. WKY-NCG: 0.9% saline (*n* = 6), SHR-NCG: 0.9% saline (*n* = 6), SHR-LPG: low dose BGP group-100 mg/kg BW (*n* = 6), SHR-HPG: high dose BGP group-200 mg/kg BW (*n* = 6), SHR-PCG: captopril-10.0 mg/kg BW (*n* = 6); * and ** indicate significant difference (*p* < 0.05) and extremely significant difference (*p* < 0.01) from SHR-NCG group. # and ## indicate significant difference (*p* < 0.05) and extremely significant difference (*p* < 0.01) from SHR-PCG group. ++ indicates an extremely significant difference (*p* < 0.01) from the WKY-NCG group. ns indicates no significant difference between different groups (*p* > 0.05).

The heart weight, the left ventricular weight, and the HE staining were used to evaluate the degree of myocardial hypertrophy and myocardial histological changes in SHR rats. After the 10-week oral administration treatment, the hearts of the experimental animals were removed and the HMI, LVWI, left ventricular wall thickness, and diameter of myocardial fiber dimension were measured to evaluate the changes in the overall heart and left ventricular mass (Figure 1A,C,D). The HMI, LVWI, the left ventricular wall thickness, and the diameter of myocardial fiber dimension of SHR rats (SHR-NCG) were significantly higher than those of WKY rats (WKY-NCG) (*p* < 0.01), which were the evidence of myocardial hypertrophy in SHR rats. High-dose BGP treatment (SHR-HPG) and captopril treatment (SHR-PCG) significantly normalized the HMI and LVWI of SHR rats (Figure 1A) while the effect of high-dose BGP on rat HMI was greater than that of low-dose BGP. The left ventricular wall thickness and the diameter of myocardial fiber dimension of rats in the high-dose BGP group and captopril treatment group were significantly lower than those in the SHR control group (SHR-NCG, *p* < 0.05). However, the left ventricular wall thickness and the myocardial fiber diameter of rats in the low-dose BGP group were not statistically different from those in the SHR control group.

The degree of myocardial fibrosis in SHR rats was visualized and assessed by Masson staining. As shown in Figure 1C, the degree of myocardial fibrosis in SHR rats (SHR-NCG) was significantly higher than that in WKY rats (WKY-NCG), while BGP treatment (SHR-LPG and SHR-HPG) reduced the degree of myocardial fibrosis in SHR rats. The percentage of rat heart fibrosis area was measured and analyzed by Image-Pro Plus (Figure 1E). The results showed that the percentage of heart fibrotic area in the high-dose BGP treatment group was significantly lower than that of the SHR control group (*p* < 0.05). However, there was no statistical difference in the area of myocardial fibrosis in the low-dose BGP treatment group compared with the SHR control group. The levels of hypertrophic markers BNP and ANP in SHR rats (SHR-NCG) were significantly higher than those in WKY rats (WKY-NCG) (*p* < 0.01), whereas high-dose BGP treatment significantly reversed the levels of BNP and ANP in the myocardial tissue of SHR rats (Figure 1B). In addition, low-dose BGP had no significant effect on the expression levels of BNP and ANP in SHR rats. These data indicate that long-term oral administration of high-dose BGP could effectively reduce myocardial hypertrophy and fibrosis in SHR rats.

### 3.2. Comparison of Proteomics Profiles by Label-Free MS/MS

**Figure 2:**
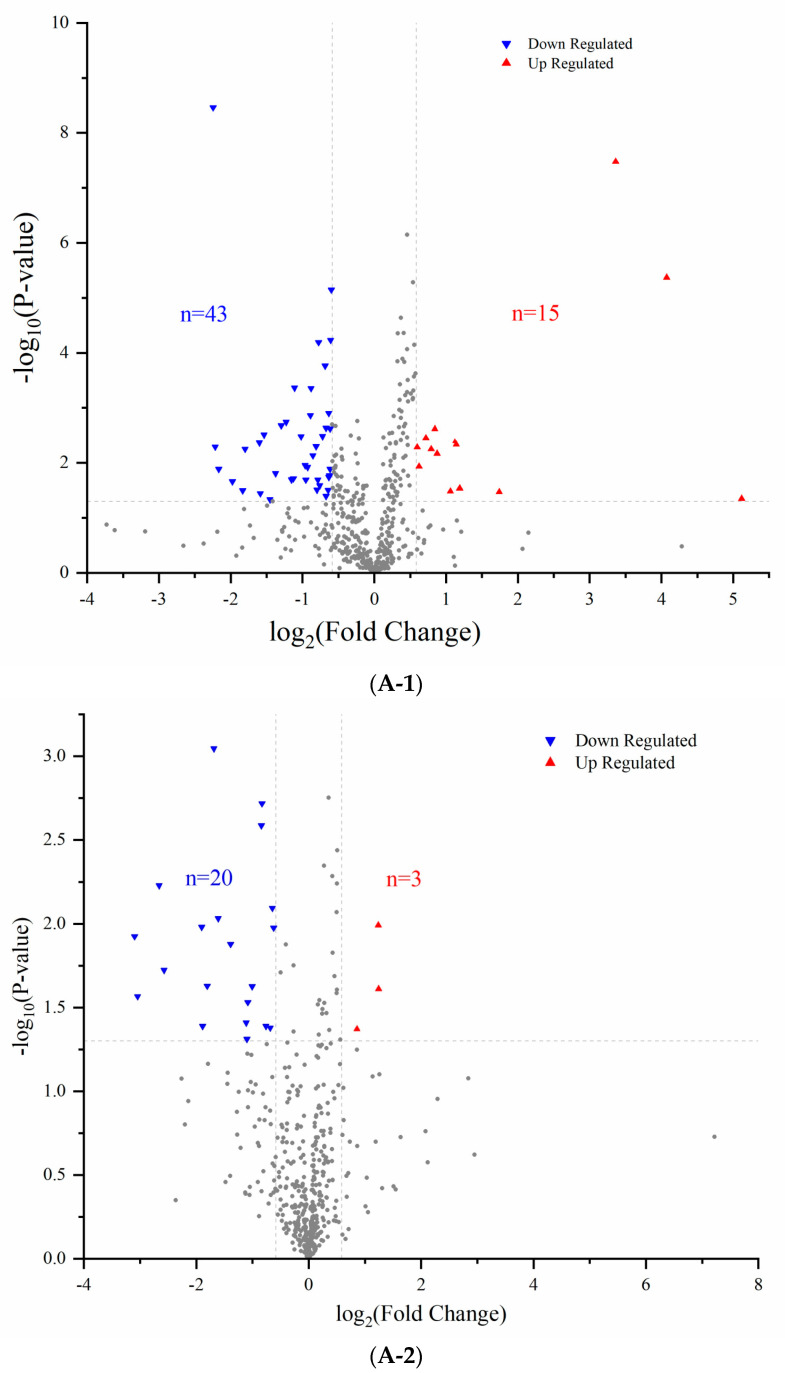

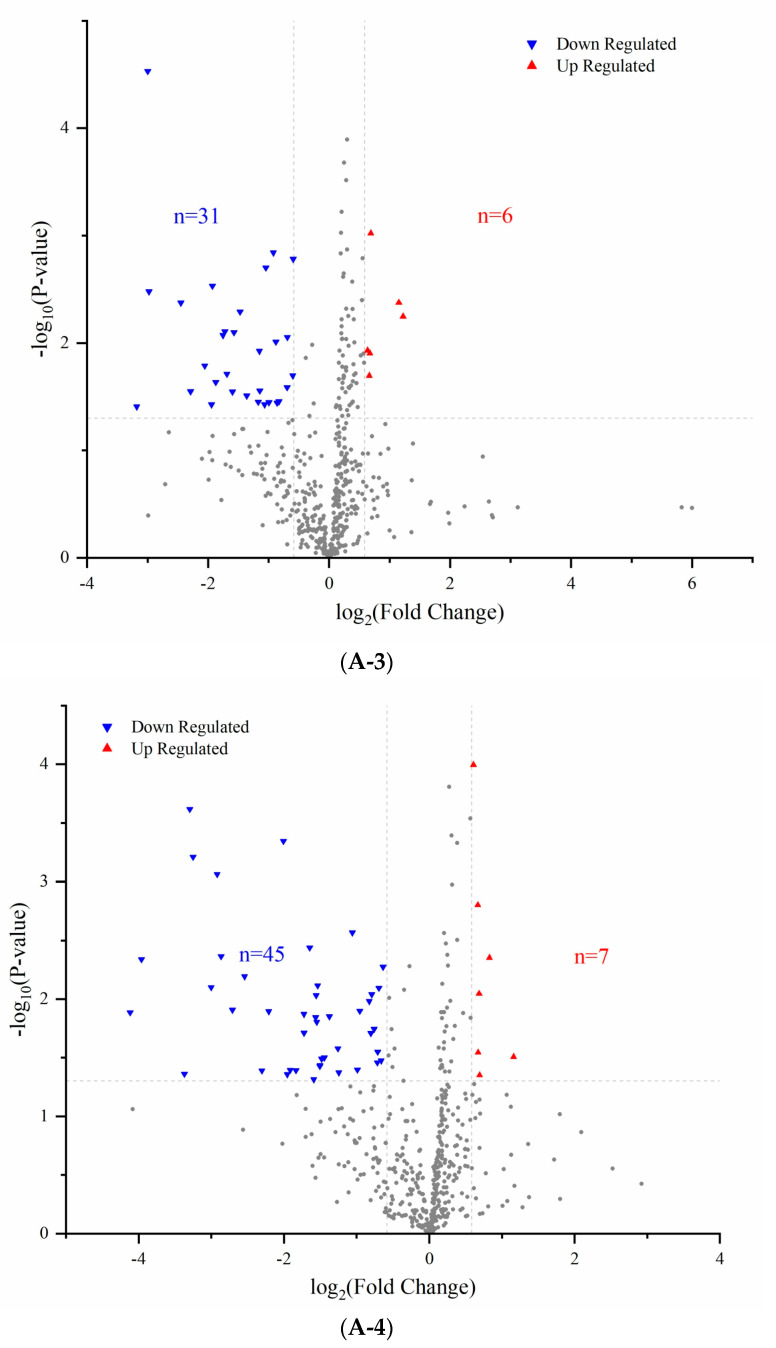

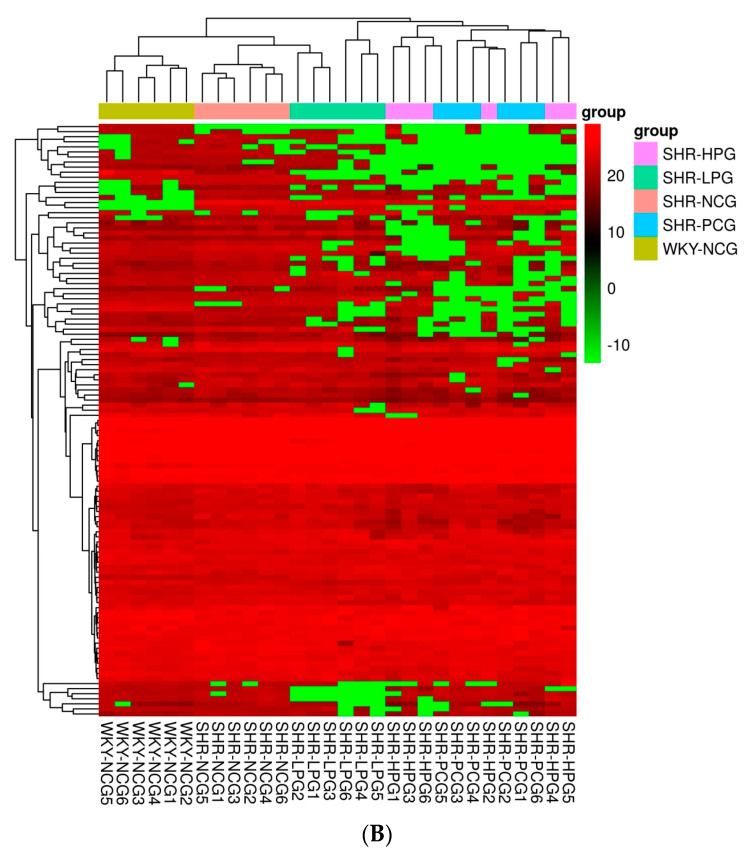
Volcano plots (**A**) and hierarchical cluster analysis (**B**) of differentially expressed proteins in rats identified by label-free proteomics. Note: Volcano plots: Compared with SHR-NCG, the differentially expressed proteins in WKY-NCG (**A-1**), SHR-LPG (**A-2**), SHR-HPG (**A-3**), and SHR-PCG (**A-4**) groups identified by label-free proteomics; The gray points in image (**A**)(**A-1**–**A-4**) represent proteins with insignificant differences. WKY-NCG: 0.9% saline (*n* = 6), SHR-NCG: 0.9% saline (*n* = 6), SHR-LPG: low dose BGP group-100 mg/kg BW (*n* = 6), SHR-HPG: high dose BGP group-200 mg/kg BW (*n* = 6), SHR-PCG: captopril-10.0 mg/kg BW (*n* = 6).

**Figure 3:**
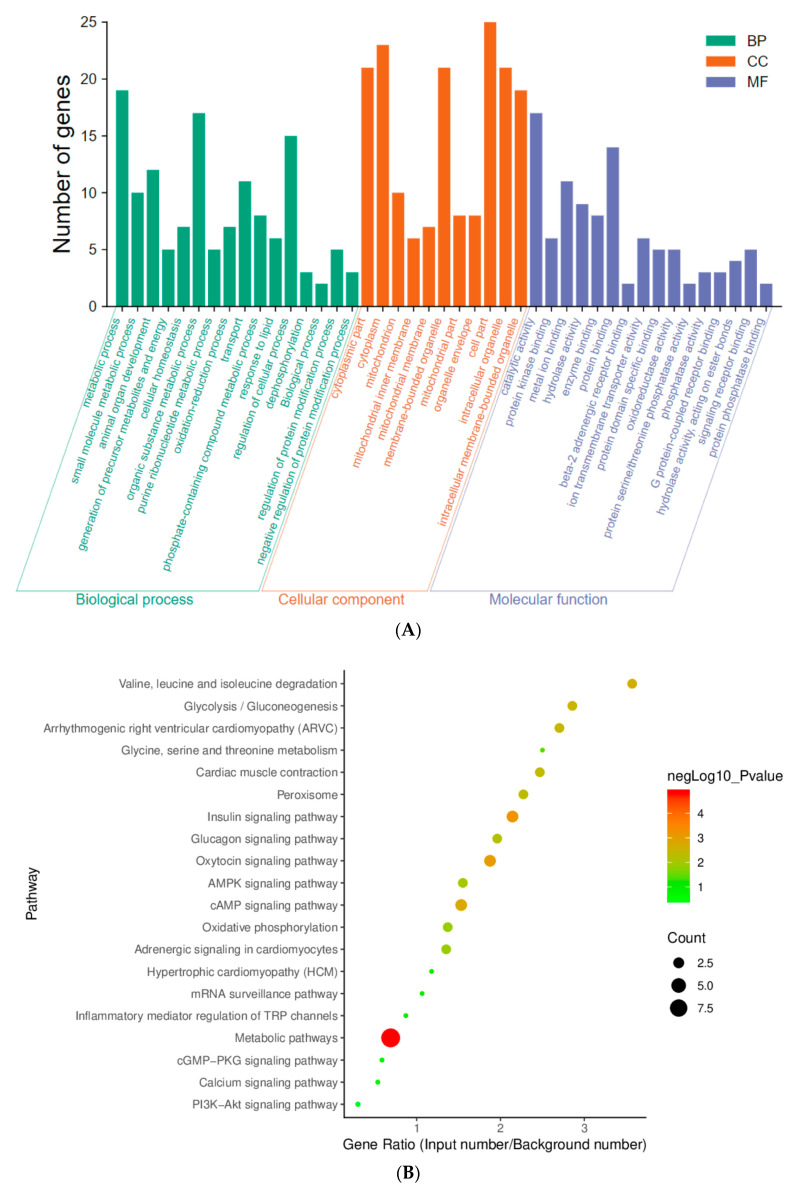

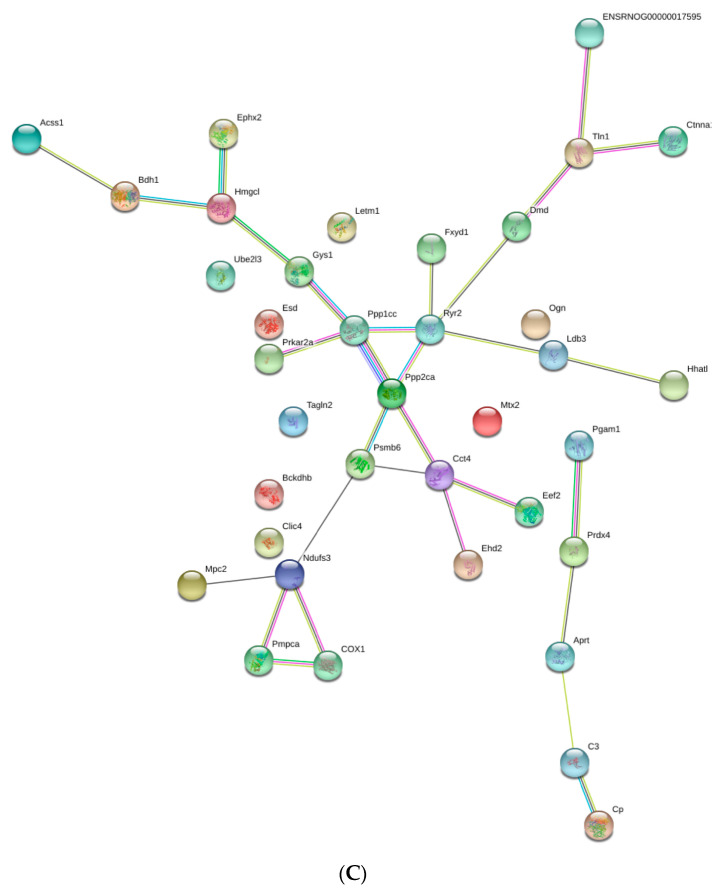
GO annotation analysis and signal pathway analysis of differentially abundant proteins in the high-dose BGP group. Note: (**A**) Go annotation analysis, (**B**) KEGG pathway analysis, (**C**) Protein-protein interaction analysis (PPI).

**Figure 4:**
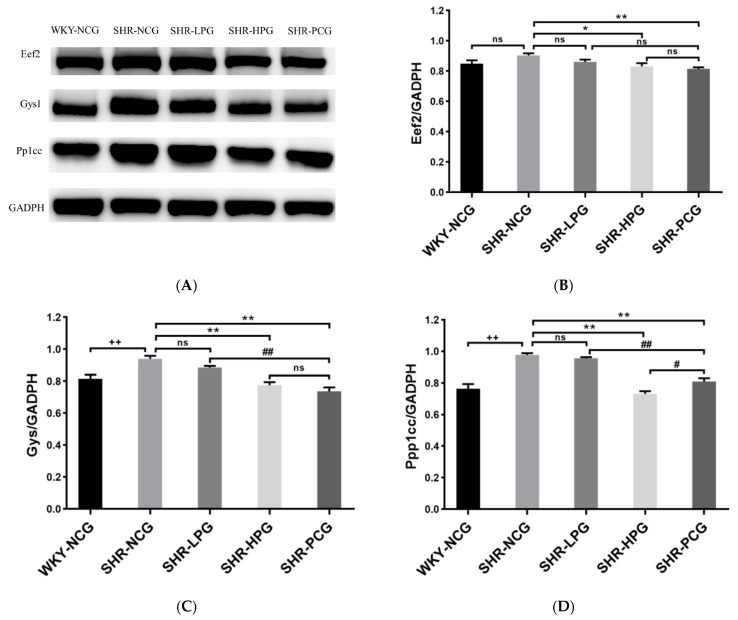
Expression levels of differentially expressed proteins of Ppp1cc (**B**), Gys1 (**C**), and Eef2 (**D**) in rat myocardial tissue. Note: (**A**) The protein bands (Eef2, Gys1, and Pp1cc) in Western blot analysis; WKY-NCG: 0.9% saline (*n* = 6), SHR-NCG: 0.9% saline (*n* = 6), SHR-LPG: low dose BGP group-100 mg/kg BW (*n* = 6), SHR-HPG: high dose BGP group-200 mg/kg BW (*n* = 6), SHR-PCG: captopril-10.0 mg/kg BW (*n* = 6); * and ** indicate significant difference (*p* < 0.05) and extremely significant difference (*p* < 0.01) from SHR-NCG group. # and ## indicate significant difference (*p* < 0.05) and extremely significant difference (*p* < 0.01) compared to SHR-PCG group. ++ indicates an extremely significant difference (*p* < 0.01) from the WKY-NCG group. ns indicates no significant difference between different groups (*p* > 0.05).

At present, proteomics has been widely used to understand the occurrence and development of diseases and the exploration of drug pathways [19]. In order to further evaluate the effect of long-term administration of BGP on the proteomics changes of SHR myocardial proteins, label-free proteomics was applied to determine the protein evolution profiles in the left ventricular myocardium of rats. A total of 529 proteins (homologous proteins grouped together) and 3892 polypeptide fragments were identified from 5 groups of rats (FDR ≤ 0.01, Supplementary Table S1). In the current study, we mainly focused our analysis on proteins with rich differences (FC > 1.5 or <0.67 and *p* < 0.05) in order to further evaluate the differences in the levels of rat myocardial protein using the SHR-NCG group as the control group. Compared with SHR rats, 58 differentially expressed proteins were identified in left ventricular myocardium in WKY rats including 43 down-regulated and 15 up-regulated proteins (Figure 2A-1, Supplementary Table S2). In the group of SHR-LPG, only 23 differentially expressed proteins were found, including 20 down-regulated and 3 up-regulated proteins (Figure 2A-2, Supplementary Table S3). However, significant changes in the expression of 37 and 45 proteins were detected in the SHR-PCG and SHR-NCG groups, respectively (Figure 2A-3,A-4, Supplementary Tables S4 and S5). These results indicated that the effects of high-dose BGP and captopril treatment on left ventricular myocardial protein expression in SHR were higher than that of low-dose BGP. All differentially expressed proteins in the five groups were used for hierarchical cluster analysis to better observe the differences in protein abundance among the groups and the analysis results were expressed in the form of heat maps. As shown in Figure 2B, the samples in the WKY-NCG, SHR-LPG, SHR-HPG, and SHR-PCG groups all had a clear dividing line with the SHR-NCG group samples. The samples in the control group (SHR-NCG) were enriched into a cluster and separated from SHR-LPG and SHR-HPG groups, indicating that BGP had a certain effect on the evolution of left ventricular protein in SHR rats. It is worth noting that the hierarchical clustering analysis did not separate the SHR-PCG and SHR-HPG groups well and neither of them formed a separate cluster.

### 3.3. GO Annotations Analysis

The above results indicated that the therapeutic effect of high-dose BGP treatment on left ventricular protein evolution in SHR rats was similar to captopril while it was higher than that of low-dose BGP treatment. To further understand and explore the functions, pathways, and interactions involved in the differentially expressed proteins (including 31 down-regulated proteins and 6 up-regulated proteins) of the high-dose BGP treatment (SHR-HPG) and the control group (SHR-NCG), we performed GO, KEGG, and PPI analyses. GO annotations were divided into three parts including biological processes, cellular components, and molecular functions (Figure 3A). In the biological processes part, the differentially expressed proteins were mainly involved in the metabolic process (small molecule, organic substance, purine ribonucleotide, and phosphate-containing compounds), animal organ development process, cellular homeostasis process, oxidation-reduction process, dephosphorylation process, and protein modification process. Cell component analysis showed that the differentially expressed proteins were found in the cytoplasm, mitochondrion, mitochondrial membrane, membrane-bounded organelle, and intracellular organelle. With regard to the molecular functional analysis, the differentially abundant proteins played an important role in catalyzing protein (ion transmembrane transporter) and enzyme (hydrolase, oxidoreductase, phosphatase) activities, and regulating protein receptor binding (beta-2 adrenergic receptor binding, G protein-coupled receptor binding, and signaling receptor binding) and enzyme (protein kinase binding and protein phosphatase binding) binding.

### 3.4. Pathway Analysis

KEGG pathway analysis can be used to explore specific biological events by analyzing the functional information of proteins in the metabolic process. KEGG pathway analysis (Figure 3B) indicated that the differentially expressed proteins in the high-dose BGP group (SHR-HPG) were related to multiple signaling pathways such as insulin signaling pathway, cAMP signaling pathway, glycolysis/gluconeogenesis, arrhythmogenic right ventricular cardiomyopathy (ARVC), AMPK signaling pathway, oxidative phosphorylation, adrenergic signaling in cardiomyocytes, calcium signaling pathway, and Pi3k/Akt signaling pathway. Under normal circumstances, proteins do not function alone instead they interact with other proteins to perform various functions in the pathway. Based on the GO function annotation analysis and KEGG analysis of differential proteins, 37 differentially expressed proteins identified in the high-dose BGP group were analyzed using STRING software (http://string-db.org/, accessed on 1 November 2020). The protein interaction network of differential changes in the left ventricle of the SHR-HPG group was further explored (Figure 3C). For the proteins contained in the central network, Ppp1cc, Ppp1ca, Ryr2, Gys1, and Eef2 were closely related to cAMP, AMPK, cardiomyocyte adrenergic signaling pathway, calcium signaling pathway, oxidative phosphorylation and messenger RNA monitoring and other pathways. The central network contained proteins that were closely related to protein binding (Dmd, Ehd2, Ppp1cc, Ppp2ca, Prkar2a, Dmd, Eef2, Fxyd1, and Ppp2ca), ion binding (Aprt, Bdh1, COX1, Cct4, Cp, Dmd, Eef2, Ehd2, Ephx2, Hmgcl, Letm1, Pmpca, Ppp1cc, Ppp2ca, Prkar2a, and Ryr2), enzyme activity mediation (Eef2, Ehd2, Ephx2, Esd, Pgam1, Pmpca, Ppp1cc, Ppp2ca, and Psmb6), transmembrane transport (COX1, Clic4, Fxyd1, Letm1, Mpc2, and Ryr2) and receptor binding (C3, Ppp2ca, and Prkar2a). These protein interactions play an important role in regulating various biological processes. For example, the interaction of Ppp1cc, Pp2ca, Ryr2, and Gys1 could simultaneously participate in multiple signal transduction pathways (cAMP, cardiac adrenaline signaling pathway, and phosphatidylinositol 3-kinase-dependent pathway (Pi3k/Akt pathway), messenger RNA monitoring and insulin signaling pathways to change homeostasis and trigger a series of complex physiological and biochemical reactions eventually leading to cardiovascular disease.

### 3.5. Western Blotting Validation

According to the results of GO annotation analysis, KEGG pathway analysis and PPI protein interaction analysis, three proteins (Ppp1cc: Insulin signaling pathway, cAMP signaling pathway, vascular smooth muscle contraction and others; Gys1: Pi3k/Akt signaling pathway, Eef2: AMPK signaling pathway), which were located in the center of the PPI interaction map and significantly related to cardiovascular disease pathways, were selected for western blot verification. The western blot results of three validated proteins (Figure 4) were the same as the results of the label-free proteomics analysis. The SHR-HPG group had significantly lower protein expression levels of Eef2 (*p* < 0.05, Figure 4B), and Ppp1cc and Gys1 (*p* < 0.01, Figure 4C,D) than the SHR-NCG groups.

### 3.6. Levels of Pi3k/Akt Signaling Pathway and TGF-β1 in SHR Rats

**Figure 5:**
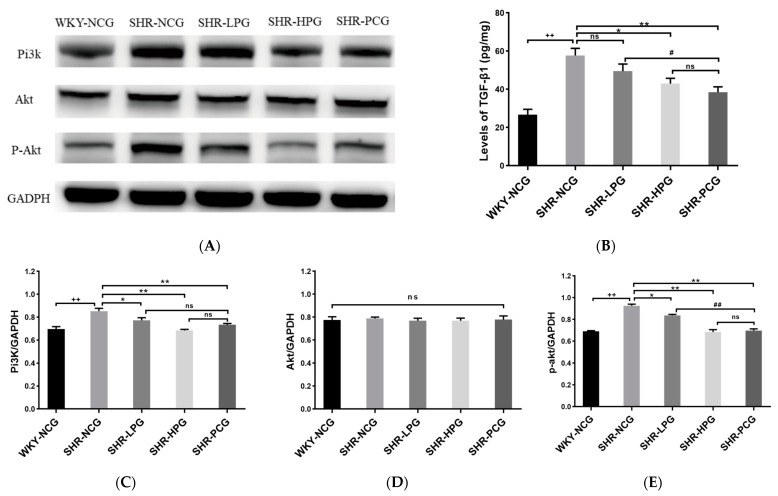
Effect of long-term oral administration of BGP on Pi3k/Akt signaling pathway and TGF-β1 level in SHR rats. Note: (**A**) The protein bands (Pi3k, Akt, and p-Akt) in Western blot analysis; (**B**) expression levels of TGF-β1; (**C**) expression levels of Pi3K; (**D**) expression levels of Akt; (**E**) expression levels of p-Akt; WKY-NCG: 0.9% saline (*n* = 6), SHR-NCG: 0.9% saline (*n* = 6), SHR-LPG: low dose BGP group-100 mg/kg BW (*n* = 6), SHR-HPG: high dose BGP group-200 mg/kg BW (*n* = 6), SHR-PCG: captopril-10.0 mg/kg BW (*n* = 6); * and ** indicate significant difference (*p* < 0.05) and extremely significant difference (*p* < 0.01) compared to SHR-NCG group. # and ## indicate significant difference (*p* < 0.05) and extremely significant difference (*p* < 0.01) compared to SHR-PCG group. ++ indicates an extremely significant difference (*p* < 0.01) from the WKY-NCG group. ns indicates no significant difference between different groups (*p* > 0.05).

ELISA and western blotting methods were used to explore the effects of BGP on the expression of TGF-β1, Pi3k, Akt, and p-Akt in the myocardial tissue of SHR rats (Figure 5). ELISA results showed that BGP treatment could effectively inhibit the over-expression of TGF-β1 in the myocardial tissue of SHR rats (Figure 5B). It was worth noting that the high-dose BGP group significantly reduced the expression level of TGF-β1 in SHR rats (*p* < 0.05), while there was no statistical difference between the low-dose BGP group and the control group (SHR-NCG). At the same time, western blotting verification results showed that, compared with the blank control group (SHR-NCG), the expression level of Pi3k protein in the SHR-HPG group was also significantly reduced (*p* < 0.01, Figure 5C). However, no significant difference was found in the expression level of Akt in the myocardial tissue of rats in each group (*p* > 0.05, Figure 5D). Fortunately, long-term oral administration of BGP significantly inhibited the expression of p-Akt in the myocardial tissue of SHR rats (Figure 5E). Furthermore, long-term oral high-dose BGP showed a more significant effect on reducing the expression of p-Akt in SHR rats than low-dose BGP. These results indicated that BGP treatment could significantly inhibit the expression of major proteins in the Pi3k/Akt signaling pathway in the myocardial tissue of SHR rats suggesting a positive effect of BGP on reversing cardiac fibrosis in SHR rats.

## 4. Discussion

At present, the causes of left ventricular hypertrophy and myocardial fibrosis include five main factors: (1) hypertension [20]: the left ventricle adapts to long-term elevated blood pressure through reactive changes, which ultimately leads to myocardial hypertrophy and myocardial fibrosis; (2) neurohumoral factors [8]: including sympathetic nervous system, RAAS system, endothelin, and others; (3) inflammatory factors [21]: T lymphocytes and mast cells; (4) oxidative stress [21]: such as H_2_O_2_, NO,·OH, O_2_-, and others; (5) related cytokines [22]: TGF-β1, insulin-like growth factor, fibroblast growth factor, and others. Among them, long-term hypertension can trigger ventricular remodeling characterized by myocardial hypertrophy [20]. In short, the long-term increase in blood pressure leads to the gradual increase of left ventricular load and eventually causes the left ventricle to enlarge or thicken, which in turn leads to cardiac fibrosis and heart failure [23,24]. It is well known that SHR rats with essential hypertension are one of the most widely used strains in cardiovascular disease studies. Therefore, SHR rats were selected as experimental animals in this study to explore the effects of BGP on myocardial hypertrophy and fibrosis caused by long-term hypertension. In addition, WKY rats with normal blood pressure were selected as a control group. 

The results in the current study indicated that the HMI and LVWI values of WKY rats (WKY-NCG) were significantly lower than those of SHR rats (SHR-NCG) after 10 weeks of intragastric administration (*p* < 0.01, Figure 1A). In addition to myocardial cell proliferation and myocardial hypertrophy, another important pathological feature of myocardial hypertrophy is the fibrosis of the extracellular matrix [25]. After HE staining and Masson staining, we found that the left ventricular wall thickness, MFD, and myocardial fibrosis area of SHR rats were significantly increased compared to WKY rats (*p* < 0.01, Figure 1C–E). These results indicate that the 20-week-old SHR rats developed obvious symptoms of myocardial hypertrophy and fibrosis. Fortunately, after 10 weeks of high-dose BGP treatment, the myocardial hypertrophy and fibrosis in SHR rats (SHR-HPG) were significantly normalized (*p* < 0.05). In addition, the increase of ANP has a unique predictive value for left ventricular dysfunction and BNP is an important monitoring indicator for patients with left ventricular dysfunction [26,27]. Fortunately, high-dose BGP significantly inhibited the over-expression of ANP and BNP in SHR rats suggesting the improvement of rat cardiac hypertrophy. The above experimental results showed that the symptoms of myocardial hypertrophy and myocardial fibrosis in SHR rats could be alleviated by intragastric administration of high-dose BGP. 

Abnormal metabolism, structure, and function of the heart are the basis of myocardial hypertrophy and myocardial fibrosis. Abnormalities that accompany myocardial hypertrophy include changes in glycolytic metabolism, calcium processing, systolic or diastolic dysfunction, disorganization of the sarcomere, abnormal expression or function of ion transporters, and changes in calcium handling [28]. Using label-free proteomics technology, we found that the protein expression of the left ventricular myocardium in SHR rats was significantly different from that in WKY rats, and long-term gavage with BGP changed the protein expression of the left myocardial myocardium of SHR rats. Interestingly, the effect of high-dose BGP on protein expression in the left ventricular myocardium of SHR rats is similar to that of captopril. Differentially expressed proteins in the myocardial tissue of the SHR-HPG group contributed to the normalization of metabolism, cell structure, cell homeostasis, animal organ development, protein kinase binding, ion transmembrane transporter activity, and G protein-coupled receptor binding in SHR rats. Notably, the regulation of G protein-coupled receptor binding in the molecular function module can play an anti-hypertrophy effect by regulating the complex network pathways that are involved in the growth of cardiomyocytes (such as the mitogen-activated protein kinase pathway, AMPK pathway, Pi3k-dependent pathway, and calcineurin-dependent pathway) [29,30,31,32]. At the same time, the KEGG analysis indicated that the down-regulated proteins in the myocardial tissue of the SHR-PCG group were involved in multiple signal pathways related to the regulation of cardiac hypertrophy and fibrosis such as the cAMP signaling pathway, cardiomyocyte adrenergic signaling pathway, calcium signaling pathway, and Pi3k/Akt signaling pathway [7,33,34], whereas the up-regulated proteins were not found to be related to signal pathways. In summary, these results indicate that BGP can play a cardioprotective effect on SHR rats through targeted regulation of multiple signaling pathways.

It is worth noting that the Pi3k/Akt signaling pathway was simultaneously detected by GO and KEGG analysis. It is generally believed that adult cardiomyocytes are terminally differentiated, so the enlargement of the heart during the process of pathological myocardial hypertrophy is mainly due to the increase in the volume of a single cardiomyocyte rather than an increase in the number of cells [35]. Therefore, the key target of potential anti-myocardial remodeling therapy is to inhibit the pathway that leads to the myocardial hypertrophic phenotype itself [28,36]. Pi3k/Akt signal pathways play an important regulatory role in the development of pathological myocardial hypertrophy [7]. Long-term overexpression of the Pi3k/Akt signaling pathway can cause pathological myocardial hypertrophy and cardiac systolic disorder [37]. High-dose BGP treatment significantly reduced the expression level of Pi3k in SHR rats and significantly inhibited the phosphorylation of Akt. It is worth noting that the activation of Akt (phosphorylated Akt, p-Akt) has a two-sided effect on the heart [38]. Physiological myocardial hypertrophy caused by the short-term activation of Akt is completely reversible with the disappearance of interstitial fibrosis and has no effect on the contractile function of the heart. However, pathological myocardial hypertrophy caused by long-term activation of Akt is accompanied by interstitial fibrosis and cardiac systolic dysfunction, which will eventually induce heart failure. In transgenic mouse models, the overexpression of Akt in mouse cardiac tissue could lead to the deterioration of its cardiac function [38,39]. Other studies have found significantly higher levels of p-Akt in patients with heart failure. After adjuvant therapy, the patient’s cardiac function was significantly recovered and the expression level of p-Akt was concomitantly significantly decreased [40]. Therefore, it is inferred that the blocking effect of BGP on the Pi3k/Akt signaling pathway is of great significance in alleviating myocardial hypertrophy and fibrosis in SHR rats.

TGF-β1, a fibrotic growth factor, is recognized as an important target for the treatment of myocardial fibrotic [22]. Its high expression can increase the synthesis of myocardial interstitial protein and induce myocardial fibrosis. Negative regulation of TGF-β1 signaling has been the research focus of various cardiac fibrosis drugs [41]. In the current study, BGP had a dose-dependent inhibitory effect on the overexpression of TGF-β1 in the serum and myocardial tissue of SHR rats which further slowed down the process of cardiomyocyte fibrosis. In addition, studies have found that complex interactions between growth factors/cytokines and hormones can promote or maintain the continuous formation of fibrosis [22]. Especially, TGF-β1 and angiotensin II (Ang-II) can jointly activate mesenchymal fibroblasts and induce the expression of multiple extracellular matrix components [42]. Studies have shown that Ang II cannot induce cardiac hypertrophy and fibrosis in the absence of TGF-β1 [43]. Interestingly, Ang II was found to induce the expression of type I collagen in cardiac fibroblasts through TGF-β1 and extracellular signal-regulated kinase [44,45]. Ang II is a vasoconstrictor that is mainly catalyzed by the key enzyme angiotensin-converting enzyme (ACE) in the renin-angiotensin-aldosterone system (RAS) [13]. Long-term excessive activation of RAS is considered to be an important cause of myocardial hypertrophy and interstitial fibrosis [46]. We have previously found that BGP also had a significant inhibitory effect on the activity of ACE [13]. In view of the complex interaction between TGF-β1 and Ang-II, and the key role of Ang-II in the RAS system. Therefore, we speculate that BGP can also play an anti-cardiac fibrosis effect by inhibiting or changing the synergistic effect of TGF-β1 and RAS system in SHR rats, but its specific mechanism needs to be further studied.

## 5. Conclusions

**Figure 6:**
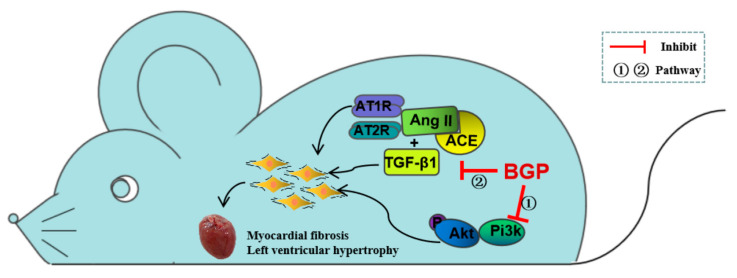
The signal pathways of BGP preventing myocardial hypertrophy and myocardial fibrosis in SHR rats.

Taken together, our research results indicated that BGP changed the proteome of myocardial tissue in SHR rats and reduced myocardial hypertrophy and cardiac fibrosis in SHR rats through the combined action of multiple signaling pathways. The inhibitory effect of BGP on the Pi3k/Akt signaling pathway and TGF-β1 in rat myocardial tissue played an important role in reducing or reversing myocardial hypertrophy and myocardial fibrosis in SHR rats (Figure 6). Our findings suggested that BGP could be potentially used as a new treatment strategy or a functional food additive to prevent and treat myocardial hypertrophy and myocardial fibrosis caused by hypertension. 

## Data Availability

Data are contained within the article and supplementary materials.

## Supplementary Materials

The following supporting information can be downloaded at: https://www.mdpi.com/article/10.3390/nu15245021/s1, Table S1: The identified peptides in bovine bone gelatin hydrolysate by Nano-LC-MS/MS (MW < 3 KDa); Table S2: List of all proteins identified by label free in this study; Table S3: List of all proteins identified by label free in this study; Table S4: List of differentially abundance proteins in the comparison between SHR-LPG and SHR-NCG; Table S5: List of differentially abundance proteins in the comparison between SHR-HPG and SHR-NCG; Table S6: List of differentially abundance proteins in the comparison between SHR-PCG and SHR-NCG.
