# Supplementary material for: Effects of Long-Term Administration of Bovine Bone Gelatin Peptides on Myocardial Hypertrophy in Spontaneously Hypertensive Rats
Source: Nutrients. 2023 Dec 6;15(24):5021. doi: 10.3390/nu15245021 (PMC10745459; doi:10.3390/nu15245021)
Supplement: Supplementary file 1 [file nutrients-15-05021-s001.zip › nutrients-2731317-Table S1.pdf]

Table S1. The identified peptides in bovine bone gelatin hydrolysate by Nano-LC-MS/MS (MW<3 KDa) (Cao etal., 2022).

**Peptide**	**ALC (%)**	**Source of Protein**	**Peptide**	**ALC (%)**	**Source of Protein**
PGFR	99	collagen a_3(IV)	LGDEPGPK	75	collagen a_1(III)
KGPR	99	collagen a_1(VII)	LGPGPPGLK	75	collagen a_1(VII)
MGPR	99	collagen a_1(I)	PGPGAPPGLR	75	collagen a_1(XXII)
RGPR	99	collagen a_3(VI)	AVPGQR	75	——
VGPR	99	collagen a_1(V)	EGPGAGK	75	collagen a_2(V)
PGQR	98	collagen a_1(VII)	KGEGSKGPR	75	collagen a_1(I)
GPLGSR	97	collagen a_3(VI)	PGAPFLPQE	75	——
LGPGSR	96	——	QPPFLPQE	75	——
GKRGAR	96	collagen a_1(I)	SGRGPK	75	collagen a_1(VII)
GPPGAR	95	collagen a_3(V)	LGMQPGAR	74	collagen a_2(IV)
KGHGNR	94	——	AGGMYGPK	74	——
TGLR	94	——	LGMQPGAR	74	collagen a_2(IV)
PGLGSR	93	——	MGLGPK	74	——
QPGPK	92	collagen a_1(II)	GPAATPGPK	74	collagen a_2(XI)
RGGPGSR	91	collagen a_1(I)	GPLGPPGLK	74	collagen a_1(XI)
KSGGPR	91	collagen a_5(VI)	GPLGSR	74	collagen a_3(VI)
VAPGQR	90	——	HGPGPLGPPGPR	74	collagen a_1(I)
GSPAGPR	88	collagen a_1(II)	KPGTDPGAA	74	——
LGPGER	88	collagen a_1(III)	PGLGPPGLK	74	collagen a_5(IV)
VAPGQR	88	collagen a_3(VI)	VAPGGAR	74	——
VGAGPK	88	——	LGPLLK	74	——
FGPGPK	87	collagen a_1(III)	RGFSGL	74	collagen a_1(I)
LGPGER	87	collagen a_3(VI)	GMLGPK	73	collagen a_1(V)
GPLGER	86	collagen a_3(V)	MGLGGR	73	——
GSKGPR	86	collagen a_1(I)	AALGLPGQR	73	collagen a_1(XVI)
VAPGER	85	collagen a_1(I)	KATMPGPK	73	collagen a_1(XVI)
GLPGQR	85	collagen a_1(I)	LGPGEVG	73	collagen a_2(XI)
VAPGER	85	collagen a_1(I)	PGAPAATPPGPQ	73	——
AAAMPGFR	84	collagen a_1(III)	PGMGFR	73	——
GPLGPK	83	collagen a_1(II)	PGVGGPGLR	73	collagen a_1(III)
PGLPGPR	83	collagen a_6(IV)	SGPAGGVP	73	collagen a_1(XVIII)
LGPGPA	82	——	AAAPLGVA	72	——
PGAGFPGAR	82	collagen a_2(IV)	LGPQDGR	72	——
PGATGMPGFR	82	collagen a_6(IV)	NVPGPPGAP	72	collagen a_5(IV)
AAAEGMPGFR	81	collagen a_1(III)	PGLGPAGGVP	72	collagen a_1(X)
AVPGSR	81	collagen a_2(XI)	RGVGPPGSP	72	collagen a_4(IV)
LGPGQR	81	——	TGPAGFPGER	72	collagen a_5(IV)
PGPQR	81	——	TPGGDGAGPK	72	collagen a_1(I)
VAPGPK	81	collagen a_1(VII)	VADEPGPK	72	collagen a_3(IV)
DVGGPGR	81	collagen a_3(V)	CTVAGR	72	——

VAMGAGL	80	——	GPLGFPGSP	72	collagen a_2(I)
LGPGAK	80	collagen a_1(XII)	KGSGPR	72	——
LGPGGK	80	collagen a_1(XII)	PGAGDPGPK	72	collagen a_1(V)
LGPGPK	80	collagen a_5(VI)	PQPFLPQE	72	——
GPSGGVP	80	——	VGMATGR	71	——
KPCDGAGPK	80	collagen a_1(I)	AAAPTKLPGAA	71	——
LGFPGPAGPR	79	collagen a_3(IV)	FGPGER	71	Hyp
LGPGPAGGVP	79	collagen a_1(III)	GHPGPGLPPGPR	71	collagen a_1(III)
NVMPGFR	79	——	PGLQPGPVG	71	collagen a_3(VI)
PGPGPPGLR	79	collagen a_2(I)	RGFPGPK	71	collagen a_1(V)
GPMGPR	79	collagen a_1(I)	RGPGPPGAP	71	collagen a_1(I)
RFGSGL	79	collagen a_2(XI)	AALGLPGQR	71	collagen a_1(XVI)
VAPLLK	79	——	DAVGGFPGE	75	collagen a_1(VI)
FGLPGPAGPR	78	collagen a_2(I)	AGPPFLPQE	71	——
GLPGQR	78	collagen a_1(I)	GPFGPK	71	collagen a_6(IV)
GPLGQR	78	collagen a_3(V)	KPGMLGPGA	71	collagen a_3(V)
GPLQPGPR	78	——	LGPGQR	71	collagen a_3(V)
MGPGFR	78	collagen a_3(V)	PGCSLGFR	71	——
PGAGFPGAR	78	collagen a_2(IV)	RGPPGPM	71	——
PGGAALPGGK	78	collagen a_2(IV)	TCLGGR	71	collagen a_1(XI)
PGPGPPGLR	78	collagen a_2(I)	VALGLSG	71	——
AAAVPGPPGAV	78	collagen a_1(I)	GMLGGR	70	collagen a_2(IV)
FGPGPK	78	collagen a_1(III)	AAAFPGLPGSP	70	collagen a_1(VII)
GGAR	78	——	AAAVGPPGSP	70	collagen a_6(IV)
AAALPGVA	77	collagen a_2(I)	GPFGPK	70	collagen a_1(XVIII)
AAAPGPPGAP	77	collagen a_1(III)	KGNDPGPK	70	collagen a_5(IV)
LGAGPPGAR	77	collagen a_2(I)	LGPGQVG	70	——
PAGGPPGPK	77	collagen a_1(V)	PGADGFRAGPK	70	collagen a_2(I)
PGLQPGPR	77	collagen a_6(IV)	PGAERGR	70	——
PGPGSR	77	collagen a_1(XII)	PGFGAPGAR	70	——
RGPPGPM	77	collagen a_1(XI)	PGGVGPPGAP	70	collagen a_1(I)
SGPAGPR	77	collagen a_1(I)	PGMVVLPGAP	70	——
AAAFPGER	76	collagen a_1(XI)	AAVCTPGAR	70	——
AAAVGPPPGAV	76	collagen a_4(IV)	PGLGFPGSP	70	collagen a_4(IV)
FGLPGPAGPR	76	collagen a_2(I)	PLGLLK	70	——
PGAGLPGPR	76	collagen a_1(XXVII)	PPGAFLPQE	70	——
PGPGSAPPGLR	76	collagen a_1(XXII)	VALGLGS	70	——
PGLGPPGLK	76	collagen a_5(IV)	VPFGNLPGSP	70	——
SSKGPM	76	collagen a_1(V)			

Note: Red P means hydroxyproline; ALC: average local confidence
